# Some Groups of Interesting Cases of Abdominal Surgery

**Published:** 1899-09

**Authors:** Charles A. Morton

**Affiliations:** Professor of Surgery in University College, Bristol, and Surgeon to the Bristol General Hospital and to the Hospital for Sick Children and Women.


### SOME GROUPS OF INTERESTING CASES OF ABDOMINAL SURGERY.

BY

CHARLES A. MORTON, F.R.C.S. Eng.,

*Professor of Surgery in University College, Bristol, and Surgeon to the Bristol General Hospital and to the Hospital for Sick Children and Women.*

---

FROM the records of eighty-two abdominal operations [1] which I have performed, I have selected three groups of cases as of special interest and worthy of record.

GROUP A.—Cases of operation for obstruction of the bile ducts by calculi. This group does not include cases in which only cholecystotomy (or removal of stones from the gall-bladder) was performed, but only those in which the ducts were obstructed by stones.

GROUP B.—Ovarian cysts burrowing deeply into the broad ligament, and therefore without a pedicle.

GROUP C.—Cases in which collections of pus within the peritoneal cavity have been evacuated through that cavity, *i.e.*, cases in which the general cavity was not shut off by adhesions.

These are records of all the cases of the kind which have been under my care.[2]

### GROUP A.

### CASES OF OPERATION FOR OBSTRUCTION OF THE BILE DUCTS BY CALCULI.

**Case I.—Stone impacted in the cystic duct, with distension of the gall-bladder. Incision of duct and removal of stone.**

Mrs. H. was sent to me by Dr. J. Wilding, in July, 1897, with a swelling in the position of the gall-bladder, and a history of typical attacks of gall-stone colic during the preceding four years. The swelling had been first noticed during the last attack of colic, a month before I saw her. There was a smooth, hard swelling, 3 in. by 3 in.

[1] These do not include hernia, or operations on the kidney from the loin, or vaginal hysterectomy, but include all abscesses arising in connection with disease of the appendix.

[2] This paper was written in March. I have had several cases of this kind since then.

in size, exactly in the usual position of a distended gall-bladder. There was no jaundice. I advised operation, but she was not able to come into the hospital until November 23rd. In the interval she had had unusual freedom from attacks of colic, but she had one lasting twenty-four hours, and followed by jaundice. The condition of the gall-bladder swelling was the same as in July.

I operated on November 26th. The gall-bladder was greatly distended with mucus, and from it I removed three gall-stones, the size of marbles. I then felt another stone just beyond the neck of the gall-bladder in the cystic duct, but neither by manipulation with one finger in the gall-bladder and one finger outside the duct, nor by efforts to drag the stone back into the gall-bladder by forceps, could I remove it. I therefore made an incision an inch long into the cystic duct on to the stone and removed it, and then sewed up the opening with a double row of Lembert's stitches of fine silk, the second row invaginating the first. Owing to the depth at which the duct lay the manipulation was difficult. The parts were of course carefully isolated with sponges, but no bile was seen, and there appeared to be no extravasation of mucoid fluid from the opened duct. I closed the incision in the gall-bladder except at one spot which I left open for a drainage tube. One end of a strand of iodoform gauze was placed below the sutured duct, and the other end brought out at the lower angle of the wound, which was shut off by suturing the parietal peritoneum and abdominal aponeurosis across the wound. In this way the opening in the abdominal wall was divided into a lower smaller one, through which the gauze protruded, and a larger upper one, and to the edges of this, the gall-bladder was united, with a small drainage tube passed into the interior. The skin was then sutured across between the openings, and then the end of the drainage tube and the strand of gauze buried in a mass of cyanide gauze and wood wool. The gauze plug was removed the day after the operation, but the gall-bladder drainage tube was retained some days longer. On December 4th bile was found discharging through the opening into the gall-bladder where the drainage tube had been. This rapidly closed, and the patient went out quite well.

After the cystic duct had become obstructed, and the gall-bladder distended with mucus, an attack of colic occurred and was followed by slight jaundice. Where did the gall-stone come from which thus temporarily obstructed the common duct? The gall-bladder was at that time shut off by the stone impacted in the cystic duct. Possibly there was more than one stone impacted in the cystic duct, and one of them moved on down the common duct.

### Case II.—Obstruction of cystic and common duct by calculi calculi removed from the gall-bladder, and a floating stone crushed in the common duct.

S.P., aged 39, was sent to me by Dr. J. Young, on January 25th, 1898. For six months she had suffered from attacks of pain in the right hypochondrium every three weeks. They usually lasted about twenty minutes, but she had been in constant pain in the same region for a fortnight before admission. There had never been any vomiting. On admission to the hospital she was very slightly jaundiced, and a small round swelling could be detected in the position of the gall-bladder. The jaundice became much more marked directly after admission, but the pain disappeared until February 12th and then returned. The swelling in the position of the gall-bladder could not always be felt. I operated on February 17th and found the gall-bladder, the size of a hen's egg, full of mucus and containing ten small gall-stones, which I removed. I also discovered a stone which darted up and down in a dilated common duct. Owing to the great difficulty in reaching the duct in this case and the way in which the stone lay on the under surface of the lesser omentum, I was obliged to crush it between my thumb and finger rather than remove it by incision. I did not find any stone in the cystic duct. After the operation, bile discharged, from the gall-bladder drainage tube and the jaundice gradually passed off. The opening into the gall-bladder closed before her discharge, on March 21st. It re-opened, however, after her discharge from the hospital, and a little bile escaped daily. She was re-admitted at the end of April, and then the fistula closed in a week. I saw her again this month (February, 1899), and found that she had remained free from jaundice and abdominal pain, and the scar had remained sound. When she came round from the anæsthetic after the operation we found she had left hemiplegia. From this the leg slowly recovered, but the hand remains useless. Its cause was uncertain—possibly some cerebral hemorrhage during the condition of cerebral congestion caused by the ether. My dresser, Mr. Johnson, has discovered records of two other cases of hemiplegia coming on after administration of ether.

### Case III.—Obstruction of common duct by floating gall-stones: anastomosis between gall-bladder and duodenum.

Miss P., aged 62, was seen, in consultation with Dr. Emily Eberle, in August last, with a view to performing cholecystenterostomy. In the previous autumn the patient began to suffer from attacks of gall-stone colic, and she had been subject to the febrile attacks resembling ague, which are often present with impaction of a calculus in one of the bile ducts. In the early autumn Dr. Eberle had discovered a gall-stone in the motions. Before the end of 1897 Dr. Eberle ceased to attend her, and in the spring of 1898 she got much worse, jaundice became very marked, and an operation was performed. Many adhesions were found around the gall-bladder, some stones were removed from its interior, but the cause of the obstruction in the common duct was not apparent, and as she was too ill for cholecystenterostomy to be performed, a biliary fistula was established, and the bile continued to discharge externally. When she had recovered from this operation, Dr. Eberle again became her medical attendant, and fearing that the continued loss of bile was preventing her complete restoration to health, asked me to see her with a view to making a communication between the gall-bladder and the intestine, and closing the external biliary fistula, from which ten to twelve ounces of bile discharged every twenty-four hours. Diarrhœa and flatulence had been relieved by the administration of ox gall by the mouth. The patient was anxious to get rid of the annoyance of the fistula. I operated on August 17th, assisted by Dr. Eberle. The bile was so viscid I could not draw it off with the aspirating cannula passed into the fistula, and I therefore plugged the latter with iodoform gauze before making the incision. There were so many adhesions in the region of the gall-bladder and bile ducts, it took some time to reach them. On passing my finger deeply behind the duodenum, I felt in the region of the head of the pancreas a large round hard body the size of a marble. When palpating it, it slipped upwards in what was evidently a greatly dilated common duct. Dr. Eberle then grasped the duct below the stone, and I did the same above it, thus fixing it, and I was just about to incise the duct over it, when I thought it lay rather too much on the under surface of the lesser omentum, and I released my grip in order to shift it further round, when it disappeared, and I was never again able to find it; but in a prolonged search for it, I found another stone half the size, also close to the head of the pancreas. This one I could easily float upwards in the duct, and could readily have removed, but I considered it was not worth the risk of incising the duct unless the larger stone could also be found and removed. I discovered another calculus the size of a small shot, fixed, very high up near the liver. Failing to remove the cause of the obstruction, I detached the gall-bladder from the abdominal wall, and united it with a Murphy button to the duodenum. The button was not the ordinary one used for the purpose, for the half which lay in the duodenum was distinctly larger than the gall-bladder half, so that the button, when detached, could not pass into the gall-bladder instead of the duodenum. I drained the region of the anastomosis with a rubber tube and a strand of iodoform gauze, and united the incision in the abdominal wound around their protruding ends. There was only a moderate degree of prostration after the operation. She was fed by the rectum only for the first twenty-four hours, and the gauze drain was removed the day after the operation. Her progress was quite satisfactory until August 30th, when she had a severe attack of hæmatemesis, but no melæna. After this she slowly regained strength, and the drainage-tube opening was healed during the following month. The button was passed ten weeks after the operation. The old fistula-opening has discharged, every now and again, a few drops of watery fluid. She gained flesh and was able to walk a mile and a half, and has had none of the old attacks of colic or pyrexia until the beginning of this year, when she had some return of the old pain. Since the operation in August, she has only had a slight conjunctival tint of jaundice for a few days in January. But serious valvular disease of the heart has developed, and her condition in January was very critical.[1]

The fourth case of this series was published in the *Bristol Medico-Chirurgical Journal* for 1897.[2] I removed a large stone which was floating freely in the common duct, and had caused a very uncommon form of pyrexia. A very interesting series of operations on the bile-ducts has lately been published by Mr. Mayo Robson[3]; and as the surgery of the bile-ducts is considered in such an exhaustive manner in Mr. Mayo Robson's paper, I would refer the reader of these records to it for further information on the subject. The difficulty in dealing with the ducts, lying as they do at a considerable depth and amongst

[1] This patient died from heart disease on May 17th, and at the necropsy I found a stone the size of a small marble in an enormously-dilated common duct.

[2] xv. 317. [3] *Brit. M. J.*, 1898, ii. 1404.

important structures, is often very great. I have not thought it safe to close the abdominal cavity without any drain, after incision and suture of the duct, and hence in Case I. the difficulty arose as to how the gall-bladder should be drained of mucus, and the peritoneal cavity in the neighbourhood of the duct drained at the same time through the same opening in the abdominal wall. I might have drained the gall-bladder through the abdominal wound, and the region of the sutured duct by means of Rutherford Morison's method of drainage through a lumbar incision. But I do not like to make this additional lumbar wound if it can be avoided, and the plan which I adopted answered very well.

The difficulty of finding floating stones in the common duct is well shown in the last case. The patient had been previously operated on by another surgeon, and no stone discovered; and after finding the largest stone, I lost it, and could not find it again. What happened must remain uncertain. I am inclined to think it darted up into some dilated and pouched portion of the hepatic duct, and there remained. I manipulated the common duct, as one would milk a cow, and had the patient's shoulder raised to a considerable extent to try and dislodge it.

GROUP B.

### Cases of Ovarian Tumours burrowing deeply into the Broad Ligament.

Two of the cases may be called broad ligament cysts, for they lay wholly within the broad ligament; in the other case the cyst reached to the costal margin, and yet its base extended deeply into the broad ligament up to the side of the uterus. Parovarian cysts of course start in the broad ligament, but do not always burrow deeply between its layers. Paroöphoritic cysts—*i.e.*, cysts starting in the hilum of the ovary, and often containing much watery growth—do commonly burrow between its layers. So also would cysts arising in the upper segment of the canal of Gärtner. The exact origin of the cysts in these cases which I record I do not know.

The removal of an ovarian cyst, large enough to ascend into the abdomen, with a distinct pedicle and with no very important or numerous adhesions, is about as simple and easy an abdominal operation as we could well have to perform. But when there is no pedicle, when the cyst lies deeply in the pelvis, and we have to open up the broad ligament and shell it out, with the danger of injury to the ureter lying just beneath, it becomes a much more difficult and anxious proceeding.

Case I.—Mrs. S., aged 52, came under my care in the General Hospital in March, 1894, for a swelling in the lower abdomen, which she had noticed for three months. A definite swelling was visible in the centre of the lower abdomen, extending rather more to the right than the left, and from just above the pelvis to two inches from the umbilicus. It was dull all over, with resonant intestine around, softly fluctuating, and could be moved laterally with considerable freedom. Nothing abnormal could be felt *per vaginam* until the tumour was pressed down, and then it could be felt bulging at the right of the cervix.

I operated on April 2nd, and found a cyst the size of a young child's head, covered by a thin layer of peritoneum, which was reflected on the tumour from the iliac fossa, and the cyst also extended deeply into the right broad ligament, and the uterus was pushed by it over to the left side of the pelvis. It lay in contact with the right side of the uterus, and there was no pedicle. It was so soft I could not satisfactorily evacuate the clear watery fluid it contained with the ovariotomy trocar and cannula; but by pushing it well forwards against the anterior abdominal wall, I was able to guide the fluid out of the incision in the latter without any passing into the peritoneal cavity. Before the cyst was emptied, a large cyst within the one first opened had to be incised. I then found that the cyst extended very deeply into the broad ligament, and in enucleating it the ureter was exposed to view, running beneath the cyst. I divided the peritoneum over the cyst, and shelled it out, clamping and dividing strands of tissue here and there, and ligating the tissue between it and the fundus of the uterus in two places. The ovarian artery and a large vein passing into the tumour were recognised and tied separately. The uterine appendages on the left side were all buried in old adhesions. The cavity from which the cyst had been removed in the broad ligament was drained by a glass tube for several days. She made an uneventful recovery, and was discharged at the end of April. In this case, the bilocular nature of the cyst is of interest. Most cysts of the broad ligament, parovarian, and paroöphoritic cysts are unilocular.

Case II.—E. H., aged 43, was sent to me in March, 1898, by Dr. L. B. Trotter, of Coleford, with a swelling in the abdomen, of nine months' duration She had a good deal of pain in the position of the swelling at times, and some frequency of micturition.

On admission, on March 20th, there was visible fulness in the right lower abdomen, and a very soft fluctuating swelling could be felt beneath the lower part of both recti, but projecting more to the right than the left. No definite outline could be made out, and the limits of the swelling could only be defined by the fluctuation wave. Nothing abnormal was made out on vaginal examination. I operated on April 5th, and found a very soft cyst in the right broad ligament. It raised the peritoneum from the right iliac fossa, so that the cyst lay over the iliac vessels and in contact with the bladder and the side of the uterus. The only place where it was free was on its posterior aspect. It was too soft to puncture with the ovariotomy trocar, so I wedged a sponge between the cyst and the abdominal wall, turned the patient over on her side, and evacuated straw-colored serous fluid from the cyst by incision. Through the opening thus made I was able to separate the cyst-wall from its capsule of peritoneum. At the upper part the cyst-wall was very thin, but shelled out with ease, after division of some strands of connective tissue containing vessels. At its lower part the cyst was much thicker, and it extended deeply, so that I felt there was considerable risk of wounding the ureter in its removal; but by clamping and dividing various strands of tissue, and peeling the cyst away where I could, I enucleated it from the broad ligament. The cyst was unilocular, and the size of a large cocoa-nut. There was no trace of the ovary attached to it. I drained the cavity left in the broad ligament for twenty-four hours. She made an uninterrupted convalescence, and went home early in May.

Case III.—N. I., aged 40, was sent to me by Mr. J. C. Smyth, of Glastonbury, for ovariotomy, in January, 1899. Her friends had first noticed an increase in size of the abdomen early last summer, and Mr. Smyth had been called in to see her a fortnight before she came under my care, and had then discovered the abdominal tumour. She had had a good deal of abdominal pain at times. There was a fairly firm, smooth tumour, which extended from the pubes to the left costal margin. It occupied more of the right half of the abdomen than the left. There were no bosses on the surface, and no thrill on percussion, or distinct fluctuation on palpation.

I operated on January 28th. On plunging the ovariotomy trocar into the cyst, nothing came through it, but thick jelly-like material welled out round it. I dragged the cyst forwards with large forceps, and my assistant kept the cyst pushed well forward against the anterior abdominal wall. I then made a free incision into the cyst (after enlarging the incision in the abdominal wall) and drew out a large mass of this viscid material. After partly emptying the cyst in this way, I made the incision into it large enough to get my hand in, and bailed out more of the jelly from the large cyst, and numerous secondary cysts which I broke into with my fingers. In this way I was able to deliver the empty cyst, except at its base. I found it had no pedicle, but burrowed to some depth into the right broad ligament, and lay by the side of the uterus. There were no adhesions, but the peritoneum of the right iliac fossa was raised by it. I got a ligature around the reflection of peritoneum from the iliac fossa into the cyst, which contained the ovarian vessels, and another around the Fallopian tube just by the side of the uterus, and after dividing the peritoneum between these two points, enucleated the cyst out of the broad ligament, dividing strands of connective tissue here and there, and clamping and dividing some vessels. The iliac vessels were freely exposed, but I did not see or feel the ureter. A small cyst was also removed from the region of the left ovary. I washed out the abdomen with sterilised saline solution, but I do not think any of the colloid material had entered it. I drained the cavity left in the right broad ligament for forty-eight hours. There was a good deal of sickness and flatulent distension of the abdomen for some days after the operation, and the urine had to be drawn off for some considerable time.

The contents of this cyst were more like those of an ovarian adenoma; but the structure of the cyst was not like that form of ovarian tumour.

It is interesting to note that in none of these cases could the cyst be evacuated by the ovariotomy trocar and cannula in the ordinary manner, but the evacuation of their contents was effected by incision without fouling the general peritoneal cavity and setting up peritonitis. In the first two cases the cysts were too soft, and in the third the contents were too viscid, for their evacuation in the ordinary manner.

### GROUP C.

### Cases in which Collections of Pus within the Peritoneal Cavity have been Evacuated through that Cavity.

When the parietal peritoneum becomes firmly adherent to the wall of an appendicitis abscess, and we can open straight into the collection of pus without implicating the general peritoneal cavity, the proceeding is not as a rule a difficult one. There may be uncertainty as to whether we have an adherent coil of bowel beneath the adherent peritoneum, and therefore we have to work very cautiously, and work through the thickened tissue mainly with the point of a blunt instrument such as a director; but when we have first to open up the general peritoneal cavity and then evacuate offensive pus through it, it becomes a more risky and difficult business. And yet we are certainly not wise in leaving pus shut up amongst adherent coils of bowel, even though adhesion may not have fixed them to the peritoneum of the anterior abdominal wall. Too often such collections of pus leak into the general peritoneal cavity before these adhesions form, and fatal peritonitis is set up. If we carefully pack around the adherent coils forming the abscess-wall with iodoform gauze, or sponges before we break down the adhesion of the coils and liberate the pus, we shall effectually prevent contamination of the general cavity of the peritoneum at the time of operation; and by the retention of a plug of gauze for twenty-four to forty-eight hours after, we can drain the abscess sac with equal safety. Some of the cases in this series show that even if pus has been freely discharged into the peritoneal cavity from the rupture of an intra-abdominal abscess, free flushing and drainage may prevent the onset of peritonitis.

In dealing with these abscesses, the method which I adopt is as follows. Directly the mass of adherent bowel around the pus is exposed, on opening the peritoneal cavity over it, it is very carefully isolated by packing around it with iodoform gauze or sponges. I then look for a spot on the surface of the mass where I think I can break through the adhesions between the coils and reach the interior; here I gently work with the end of a director and my finger-tip. Presently a little, usually offensive, pus escapes, and is quickly caught on a sponge. The opening is enlarged by the finger, the pus caught on sponges as it wells up, and then when it ceases to flow the cavity is plugged with a strand of iodoform gauze so that no pus can escape. The surrounding mass of gauze, or the sponges, are then removed, and the neighbouring coils examined, by inspection and gentle sponging, to see if any pus has found its way amongst them. In my own cases, the gauze packing has always prevented this. If a little pus had escaped amongst the coils of bowel, it would be sponged up; if anything like free escape of pus into the general peritoneal cavity had occurred, it would be flushed out with boiled saline solution. A fresh strand of gauze is then packed around the mass, and the end brought out of the incision in the abdominal wall. The piece of gauze within the abscess cavity is then replaced by a large glass drainage tube, in which lies a strand of iodoform gauze to act by capillary attraction. The general peritoneal cavity is thus shut off while the abscess sac is drained. Sometimes I have used the wick drain, introduced by Morris of New York, instead of the glass one. This consists of a strand of gauze, surrounded by green protective, just as the tobacco is surrounded by the paper in a cigarette. The gauze acts as a capillary drain, and the green protective prevents adhesion to surrounding tissues. Usually an anæsthetic is required for a few minutes when the gauze is removed from around the suppurating mass, forty-eight hours after the operation. By this time, and probably sooner, protective adhesions will have shut off the general peritoneal cavity around the gauze-plugging. The adhesion of the gauze to the peritoneum is so firm that it often requires some force to dislodge it, and hence the need for an anæsthetic.

Only a certain number of the cases in this group have been cases of appendicitis. Two are cases of suppuration from perforation of the bowel, one in a malignant growth and one from a fish-bone. One is a very interesting case of abscess in the mesentery from localised necrosis. Fæcal matter, as well as pus, was flushed out of the general peritoneal cavity in one case, which recovered without peritonitis.

The cases in this group are too numerous to give their histories, and I have had to be content with a brief outline of the operations and results. In the first seven cases, the abscess probably arose in connection with disease of the appendix. In most of the cases, the discovery of the perforated appendix, or an appendix concretion in the pus, placed their origin beyond doubt, and the origin of the others from the appendix was extremely probable.

CASE I.—W., aged 22. There was a collection of pus shut in by adherent coils around a sloughing appendix. On opening the peritoneal cavity, a mass of adherent intestine was found lying just internal to, and slightly below, the cæcum, which was adherent to it, and overlapped it to a slight extent. There were no adhesions to the anterior abdominal wall. I carefully packed sponges around it in all directions. At first I did not see where I could break into the mass and evacuate the pus, but what looked like the junction of ileum and cæcum (so smooth was it) turned out, on further examination, to be only an adhesion, and by breaking it down I liberated a few drops of pus. The pus was at once sponged up, and I then gradually broke into the abscess cavity through the adhesions. The cavity was the size of a hen's egg, and I did not feel the appendix within it, but I avoided much manipulation for fear of breaking down adhesions on the under aspect of the adherent coils, and thus allowing pus to escape into the general peritoneal cavity. After removing the gauze around the mass, I plugged the abscess with a strand of iodoform gauze, and brought the end out through the incision in the abdominal wall. I also packed around this central strand of gauze with other strands, so as to shut off the peritoneal cavity, but they did not penetrate into the abscess sac. These were removed in forty-eight hours. On the fifth day after the operation a slough of the appendix an inch long was discharged from the abscess cavity, and the patient went out quite well a month later. She never had any signs of general peritonitis.

CASE II.—Wm. T., aged 8. In this case, the mass of adherent coils around the collection of pus was adherent at one place to the peritoneum of the anterior abdominal wall; but I had to open the peritoneal cavity just above, in order to see what condition I had to deal with, and in separating the adherent parietal peritoneum off the mass a little pus exuded. This was quickly sponged up, and after the opening had been enlarged with sinus forceps, I passed my finger in, the pus as it escaped being quickly absorbed by masses of gauze placed around. I did not feel the appendix, but the position of the abscess—at McBurney's point—left little doubt as to its origin. The abscess-sac was drained, and the general peritoneal cavity shut off as in the last case, with the same successful result.

CASE III.—C., boy, aged 16. In this case, on opening the peritoneal cavity, I found the mass on the outer aspect of the middle of the ascending colon, continuous with the lower end of the kidney. I plugged all round it with a quantity of iodoform gauze, and then separated adhesions, and evacuated some offensive pus and a typical appendix concretion. Before removing the temporary plugging, I inserted a strand of gauze into the abscess sac, and brought the end out through the incision in the abdominal wall. The temporary plug was then replaced by two strands of gauze, one placed on each side of the strand lying in the abscess sac, and the ends brought out through the abdominal incision. The temperature fell to normal after the operation, and there were no signs of general peritonitis; and in a month's time the boy's general condition was good, and only a sinus remained; but later on fresh abscesses (extra-peritoneal) formed and were opened, sinuses persisted, and the boy died nearly a year after the first operation. The parents refused to take the risk of an operation for removal of the diseased appendix.

The question has been much discussed during the last few years (especially by American surgeons) whether, in these cases of localised suppuration around an appendix, we should break down those adhesions on which the safety of the peritoneal cavity depends and remove the diseased part. The feeling of most surgeons, and certainly my own, is that the risk of setting up general peritonitis is too great. If the appendix is found lying loosely in the abscess sac, we should, I think, undoubtedly remove it; but we are not justified, I consider, in breaking down the adhesions between adherent coils of bowel which shut off the infected area, in order to remove it. Perhaps if we did remove the appendix in all these cases we might less often have troublesome sinuses after evacuation of an appendix abscess, but we might, I think, sacrifice the life of the patient; and these persistent sinuses are not very frequent, and only very rarely do they lead to any serious result. In the case which I now record, I advised that the appendix should be removed, when it became evident that it would cause chronic trouble, even though in the presence of septic sinuses the operation involved some risk.

Case IV.—W. H. O., aged 10. In this case the collection of pus was situated in almost the same situation as in the last—outside the middle of the ascending colon, between the colon and the parietes; but it extended from this upwards, and was more difficult to reach. I was able to plug below it with a sponge packed in at the outer side of the cæcum, but could not push gauze or a sponge in above it, under the liver. The ascending colon formed a barrier to the entrance of pus into the general peritoneal cavity on the inner side. I turned the boy well over on his right side, and then, by separating adhesions, evacuated offensive pus. Nothing was seen of the appendix; but I had little doubt the abscess arose in connection with it, though not in the usual position for an appendix abscess. After thoroughly sponging out the abscess sac, I sponged out the fossa under the liver above the abscess sac, but found no pus there. The abscess sac was drained by means of Morris's wick drain, already described. Although the pus was evacuated, the boy's general condition got worse, and he died two days after the operation. At the necropsy, it was found that the abscess cavity had not only formed a considerable swelling within the peritoneal cavity by adhesions between the outer aspect of the ascending colon and the parietes, but had also eroded the peritoneum and the anterior aspect of the right kidney. The perforated tip of the appendix lay in the abscess sac. The abscess was, of course, primarily intra-peritoneal, and yet it had eroded the peritoneum over the kidney, and had not found an exit into the general peritoneal cavity, so great is the protecting action of adhesions within the peritoneal cavity in these cases. The necropsy showed that the evacuation of the pus had been accomplished without any infection of the general peritoneal cavity, for there were no signs of any general peritonitis.

I have operated on four cases of appendix abscess situated outside the middle of the ascending colon. Two are here recorded. In the other two the general peritoneal cavity had been shut off by adhesions at the time of operating, and therefore they do not come into this group. In one of these I found an appendix concretion in the abscess; in the other only the similarity in position, and the absence of any other obvious cause, led me to diagnose perforation of the appendix as the almost certain origin. No doubt in all the appendix lay directed upwards under the ascending colon (as was demonstrated in one of the cases), and the tip perforated in that position.

Case V.—Boy, aged 16. A collection of pus was shut in by adherent coils of bowel, and omentum around a perforated appendix.

There were no adhesions to the abdominal wall. When the peritoneal cavity was opened, pus was found leaking out through a perforation in the omentum. The peritoneal cavity was washed out, but death from peritonitis occurred ten hours later. The necropsy showed that the peritonitis must have started before operation, as it was advanced in parts of the abdomen away from the focus of suppuration. An earlier operation would probably have saved the boy's life.

Case VI.—A. B., aged 20. This was a case of large pelvic abscess, of doubtful origin; but I have included it amongst the appendicitis cases, as most probably it was due to a sloughed appendix, hanging over towards the pelvis. I have record of one fatal case of peritonitis from perforation of the appendix, in which the cavity of the true pelvis was full of pus, and there was no pus around the appendix; and yet a perforation of the appendix in the iliac fossa was clearly the cause of the peritonitis, for an appendix concretion was found lying there. In other cases we find the appendix hanging over the pelvic brim, with the perforated tip in the pelvic cavity. I believe the case to have been one of this kind, in which probably not only was the appendix perforated, but much of it had sloughed off. The patient presented a definite swelling just below McBurney's point, and a large pelvic swelling bulging into the rectum. I opened the abdomen over the abdominal mass, and found that it consisted of a mass of adherent bowel and omentum, which was only adherent at one or two spots to the anterior abdominal wall. I packed around the mass with iodoform gauze, and then broke down adhesions with my fingers, and tied and divided portions of adherent omentum, until the lower extremity of the cæcum was well exposed, but I could not find the appendix. While doing this, a little pus exuded from amongst the adherent coils. I then broke down the adhesion of the bowels on the deeper aspect of the mass, and thus worked into Douglas's pouch, and then a very free discharge of horribly offensive pus took place, and I found Douglas's pouch had been converted into a large abscess cavity, pushing forward the uterus. Before opening this up, the gauze packing was removed, as it was evidently impossible to protect the general peritoneal cavity from infection by its retention. I found the right Fallopian tube and ovary greatly swollen, and suspecting that the abscess might have arisen in a ruptured pyosalpinx, I removed them; but subsequent examination showed that although the tube contained lymph, its inflammatory condition was probably due to its having formed part of the wall of the abscess sac. No trace of the appendix could be discovered, but I thought there was an ulcerated area where it might have sloughed off. I very thoroughly flushed the whole peritoneal cavity with boiled saline solution, and placed a glass drainage tube in the pelvis, with a strand of iodoform gauze within it for a capillary drainage. The general condition improved very much after the operation, and the abscess sac continued to drain well; but another intra-abdominal abscess formed, a fortnight after the operation, on the opposite side of the abdomen above the level of the umbilicus, and when the pus was evacuated the knot of a sulpho-chromic catgut ligature came away in it, showing that it arose in connection with the primary seat of suppuration. After the opening of the secondary abscess, she made a satisfactory recovery. Although the general peritoneal cavity was infected by the offensive pus at the time of the first operation, yet by thorough flushing the onset of general peritonitis was prevented.

Case VII.—Another case belonging to this group has already been published,[1] as one of recovery after draining a pyæmic abscess of the liver. A collection of pus was shut up around the diseased appendix, but there were no adhesions to the anterior abdominal wall. I removed the appendix, flushed out and drained the peritoneal cavity, and the boy did not develop any signs of peritonitis, and after evacuation of the pyæmic abscess in the liver, recovered.

**Case VIII.—Evacuation of pus from the general peritoneal cavity, and drainage of ruptured pelvic abscess: recovery without peritonitis.**

S. E., aged 36. On opening the peritoneal cavity just above the pubes, several ounces of very offensive pus escaped, and I found a collection of pus shut up in the true pelvis by adhesion over the brim, and at one spot this had given way and the pus leaked out. The rupture through the adhesion was enlarged by my finger and the pus freely evacuated, the general peritoneal cavity well flushed with boracic lotion, and the abscess drained with a glass drainage tube, within and around which I placed strands of iodoform gauze. The cause of the abscess was probably suppuration in a Fallopian tube, as she had an offensive vaginal discharge. The rupture probably occurred two hours before operation, when she was seized with very severe pain in the abdomen. The glass drainage tube and the gauze-plugging around were removed in twenty-four hours after the operation and a rubber tube substituted. As the discharge remained offensive, I had the cavity frequently irrigated, and then it became sweet. The sinus persisted for several months.

**Case IX.—Abscess in the mesentery: evacuation through peritoneal cavity without infection of peritoneum; death nine days after operation, from pneumonia.**

J. P., aged 50. In this case there was a large swelling just below the umbilicus, with high temperature. The patient had been a heavy drinker. That there was an intra-peritoneal abscess seemed certain, but the cause was very obscure. On opening the abdomen I found a hard mass, the size of an orange, lying over the lumbar spine. It was covered by adherent coils of small intestine; but the great omentum passed over it without adhering to it. I very thoroughly packed all round the mass with iodoform gauze, and then broke down an adhesion between the adherent coils on the upper part, and thick offensive pus came out and was rapidly sponged up. My finger passed into a cavity amongst the adherent coils just like an abscess cavity from perforation of the appendix. After sponging up all the pus as it exuded, I plugged the cavity with gauze, and then withdrew the surrounding mass of gauze and passed small sponges on holders in various directions into the peritoneal cavity, but found no pus there. The strand of gauze which had been inserted into the abscess sac was then replaced by a large glass drainage tube, and around this, outside the cavity, was placed a mass of gauze to shut off the general peritoneal cavity while the abscess drained. It was removed under chloroform in forty-eight

[1] *Bristol M.-Chir. J.*, 1897, xv. 323.

hours. The progress of the patient, so far as his abdominal condition was concerned, was most satisfactory; but he got lobar pneumonia, and died nine days after operation. At the necropsy the abscess cavity was found almost obliterated, and there was no trace of general peritonitis. It evidently had arisen in the mesentery, and probably from a limited necrosis, as a necrotic area was found in that structure. The appendix was normal.

### Case X.—Collection of pus beneath adherent cysts in the pelvis: evacuation through peritoneal cavity without general infection of peritoneum.

E. S., aged 25. On opening the peritoneal cavity, I found a mass of adherent omentum and bowel around a small cyst of the left ovary, and a mass of small cysts firmly adherent in Douglas's pouch. On separating some omental adhesions, some serous fluid escaped, and then on passing my finger deeply into Douglas's pouch between the adherent cysts, some thick pus welled up and was caught on sponges. I freely flushed out the whole peritoneal cavity with sterilised saline solution. I did not remove any cysts, as to get the adherent mass away would have been a serious operation with suppuration in the pelvis. I left a large glass drainage tube in the peritoneal cavity, reaching down into Douglas's pouch. This was replaced by a rubber one forty-eight hours after. A fortnight after the operation another collection of pus formed higher up in the abdomen, but after incision and drainage the cavity closed. A sinus, however, persisted from the first operation, at the time she left the hospital, about four months after operation, but the discharge from it was very slight, and I believe it healed soon after her return home.

### Case XI.—Intra-abdominal abscess in connection with a malignant growth in the bowel: drainage through the peritoneal cavity.

M. J., aged 50. The patient had a fairly large abdominal swelling to the right of the umbilicus, associated with marked pyrexia. On opening the abdomen over the swelling I came on a red hard mass, with the omentum adherent over it, and with adhesions here and there to the parietal peritoneum. On gently separating some omentum lying on it pus welled up, and this I caught on a sponge, and kept the sponge pressed against the opening until I got iodoform gauze plugged round. I then broke down the adhesions, and liberated some thick, very offensive pus. After the evacuation of the pus a portion of the swelling remained very hard. Before removing the gauze I passed a large glass drainage-tube into the abscess cavity. I then replaced the gauze by a fresh strand, the end of which was brought out of the opening in the abdominal wall with the end of the glass drainage-tube. It was removed in forty-eight hours, by which time the general peritoneal cavity had become securely shut off. Some fæcal discharge was observed on the second day. After temporary improvement, her strength failed, and she died a month after the operation. At the necropsy the abscess was found to be due to perforation of a malignant growth in the hepatic flexure of the colon. The growth was extensive, but had not blocked the lumen of the gut. The general cavity of the peritoneum had not been infected.

### Case XII.—Evacuation of pus and fæces from the general peritoneal cavity: free irrigation: recovery.

Girl, aged 8. This case is a remarkable one, and was brought before the Bristol Medico-Chirurgical Society by Dr. Michell Clarke and myself some years ago. There were signs of an intra-abdominal abscess in the left iliac fossa. In fact, the case very much resembled an appendicitis abscess, except that the abscess was on the left side. On dividing the abdominal wall on the outer aspect of the swelling, *i.e.* just inside the anterior superior spine, I came down on what seemed to be greatly thickened peritoneum, and on gradually working through this with the point of a director some pus escaped. I dilated the track gently with my finger, and found a smooth-walled cavity the size of a hen's egg, out of which was an opening at the other end, through which my fingers passed into the peritoneal cavity, where free intestine could be felt. The wall of the abscess cavity seemed to be composed of adherent intestine. The general peritoneal cavity was washed out with a large quantity of hot boracic solution through the abscess sac. As soon as the irrigation was commenced, what appeared at first to be shreds of tissue from the interior of the abscess came away with the fluid, but they were quickly recognised as bile-stained, and were undoubtedly the contents of the first portion of the small intestine. The irrigation was continued until all this material had come away, and then a large-sized drainage tube was placed in the abscess cavity as far as the internal opening into the peritoneal cavity. The fæcal discharge of the same character persisted for some weeks, but there were no signs of peritonitis, and the child was discharged from the hospital six weeks after the operation.

The doubt as to the origin of the abscess was never cleared up. It must, I think, have given way into the peritoneal cavity just before the operation, perhaps during struggling as she went under the anæsthetic, and possibly a small area in one of the adherent coils of bowel, softened by inflammation, gave way at the same time, and hence the fæcal matter found in the general peritoneal cavity.

To these cases must be added two more. One of these I have already published.[1] A large abscess formed under the meso-colon from perforation by a fish-bone, which was found in the abscess sac. I was able to stitch the surface of the meso-colon to the parietal peritoneum before evacuating the pus and thus shut off the general peritoneal cavity from infection. The other case was one of enormous suppurating hydatid of the liver. There were no adhesions between the liver and the parietal peritoneum, but by stitching the liver surface to it around a small area, I was able to evacuate pus and an enormous number of daughter-cysts without infecting the general peritoneal cavity. The case will be published later on, with other cases of hepatic surgery.

[1] *Brit. M. J.*, 1894, i. 241.

